# Identification of Mastication Organ Muscle Forces in the Biocybernetic Perspective

**DOI:** 10.1155/2015/436595

**Published:** 2015-03-26

**Authors:** Edward Kijak, Jerzy Margielewicz, Damian Gąska, Danuta Lietz-Kijak, Włodzimierz Więckiewicz

**Affiliations:** ^1^Department of Prosthetic Dentistry, Faculty of Medicine and Dentistry, Pomeranian Medical University, Rybacka 1, 70-204 Szczecin, Poland; ^2^Silesian University of Technology, Krasińskiego 8, 40-019 Katowice, Poland; ^3^Department of Dental Propedeutics and Phsysiodiagnostics, Pomeranian Medical University, Rybacka 1, 70-204 Szczecin, Poland; ^4^Department of Prosthetic Dentistry, Faculty of Dentistry, Wroclaw Medical University, 26 Krakowska Street, 50425 Wroclaw, Poland

## Abstract

*Purpose of the Paper*. This paper is an attempt to mathematically describe the mastication organ muscle functioning, taking into consideration the impact of the central nervous system.* Material*. To conduct model tests, three types of craniums were prepared: short, normal, and long. The necessary numeric data, required to prepare the final calculation models of different craniofacial types, were used to identify muscle and occlusion forces generated by muscles in the area of incisors and molars. The mandible in model tests was treated as a nondeformable stiff form.* Methods*. The formal basis for the formulated research problem was reached using the laws and principles of mechanics and control theory. The proposed method treats muscles as “black boxes,” whose properties automatically adapt to the nature of the occlusion load. The identified values of occlusion forces referred to measurements made in clinical conditions.* Results*. The conducted verification demonstrated a very good consistency of model and clinical tests' results. The proposed method is an alternative approach to the so far applied methods of muscle force identification. Identification of muscle forces without taking into account the impact of the nervous system does not fully reflect the conditions of mastication organ muscle functioning.

## 1. Introduction

While formulating the numeric model of the mastication organ, primarily passive forces and active forces affecting the mandible are defined. Passive forces assume reactions in temporomandibular joints and occlusion force, while active ones reflect muscle activity. One of the methods of muscle force identification is the solution of the issue of mastication organ biostatic balance. Additionally, the biostatic balance is defined as a situation when the mandible, loaded with any arrangement of forces, is not moved as well and does not change its orientation; that is, it remains in a stable position for any period of time. Research defined in such a way can be analysed by both static and dynamic methods. Regardless of the class of the formulated problem, proper causal relations are introduced by means of analytical or vector mechanic methods. From a theoretical point of view, two classes of identification methods of mastication organ muscle forces are distinguished. The first one is represented by methods where active muscle forces are replaced by single resultant. A detailed description of these methods is presented in the following works: Weijs, 1989, and Erhardson et al., 1993 [1, 2]. The second group includes methods whose formal basis is the direct identification of muscle forces, which are presented, for example, by Osborn and Baragar, 1985; Peck and Hannam, 2000; and Sellers and Crompton, 2004 [3–5]. In addition, we cannot forget about methods using the MES formalism, which is applicable in situations of stress and deformations of the mandible assessment. These methods are often applied during analyses of any kind of biomechanic issues of the stomatognathic system, and they are described in works by Vollmer et al., 2000; Igić et al., 2001; Iwasaki et al. 2003; Koolstra and Van Eijden, 2004; Peck et al., 2004; and Hannam et al., 2008 [6–11].

Model tests of the mastication organ are very often conducted on flat models. Reduction of the spatial structure to a flat one each time assumes a symmetrical mandible load with active and passive forces. Despite a number of attempts, a clear method has not yet been formulated, enabling precise calculation of sizes of forces generated by particular mastication organ muscles. In addition, there is no technology enabling direct registration of muscle forces and loads affecting temporomandibular joints. For this reason, the numerically calculated values of muscle forces are hard to be verified. However, computer simulations bring the expected effects when the conditions of calculation model support are correctly defined. Usually, they are adopted as fixed articulated supports, located within temporomandibular joints. This way of model support is particularly convenient when static balance equations are used. In the case of application of formalism of method of finite elements, the modelling person has an available broad selection of various ways of articular reaction reflection. Reactions occurring in the joints can be modeled, at least in three ways: (1) as a support articulated elastic; (2) by means of active forces, distributed along surface of condyloid process heads, as described by Korioth et al., 1992 [12]; (3) or by contact connection occurring between transport disks the acetabulum and condyloid process heads.

The assessment of causal relations taking place between the occlusion force and muscle forces is a difficult issue from the point of view of biomechanics. These difficulties are caused by the fact that spatial structure of the placement of muscle fibres is able to generate various ranges of statically equivalent forces of combinations ensuring biostatic balance of the mandible. However, the advantage of this diversity, replacing the solutions is the possibility to counteract unwanted biomechanical reactions. Restrictions and difficulties related to identification of forces generated by muscles were the reason to undertake activities to formulate a biocybernetic mastication organ model. In the biocybernetic perspective, muscles are considered “black boxes” whose properties adapt automatically to the nature of the load on the dental arch of the mandible. The model approach proposed in this paper is an alternative to the so-far applied methods of muscles force identification. The formal basis for the solution, defined in such a way as a research problem, is the methods that can be used in mechanics and control theory.

## 2. Methods and Material: The Formulation of the Numerical Model

**Figure 1:**
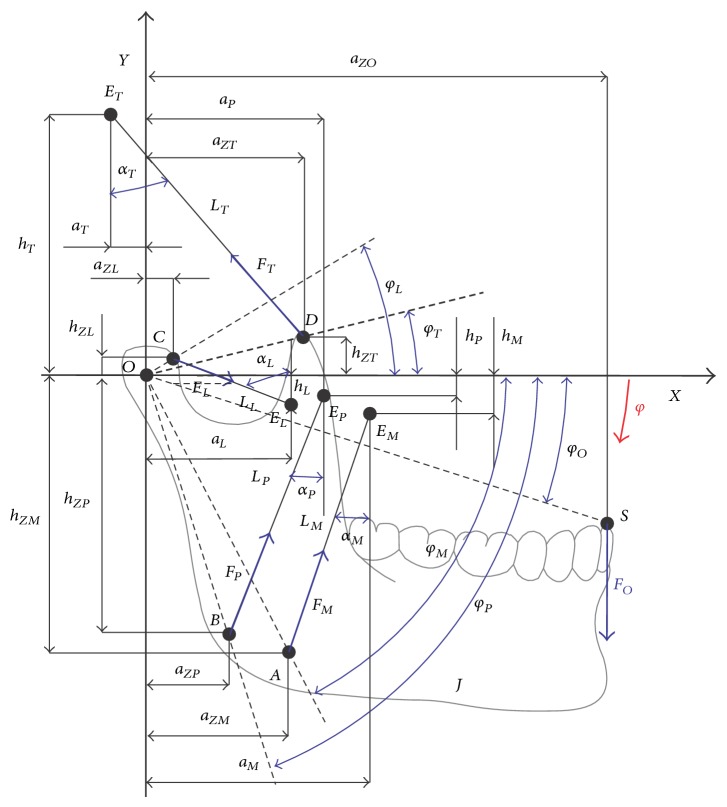
The schematic diagram reflecting the relations between the muscle system and the skeletal system of the mastication organ.

During numeric model formulation, used to identify the muscle forces of the mastication organ in the biocybernetic perspective, the following assumptions were adopted. A mathematical model was derived for the case of symmetrical dental arch load of the mandible with occlusion forces. The condyloid process heads assume a stable location in the articular space. The mandible was treated as a nondeformable rigid form. Adoption of the mandible as a rigid element is justified from the point of view of model tests, as its rigidity in comparison to muscle stiffness is much greater. In addition, during conducting numeric experiments, there is a possibility of angular movement of the mandible in relation to the axis running through the centres of condyloid process heads. The assumption is also justified, because in the initial phase of abduction mandibular movement may be described with great accuracy as “clear” rotation, the so-called pivoting movement. Additionally, limitations resulting from maximum force that particular muscles are able to generate during dental arches load have been taken into account. The force on the dental arch of the mandible changes from zero to the agreed value. Additionally, the direction of its operation is perpendicular to the horizontal axis of the coordinate system in respect of which numeric calculations were performed. Bearing in mind what the work of Ide and Nakazawa [13] stated and referring to the activity of particular muscles when generating occlusion forces in the set zones of dental arches of the mandible, additional model assumptions were made. According to the aforementioned authors, temporal muscles are inactive when occlusion forces are generated in the zone of incisors. In order to demonstrate the relevance of the information stated by the abovementioned researchers, measurements of masticator's function potentials and temporal muscles during closing dental arches in the surroundings of incisors on dynamometer covers were conducted. These preliminary tests clearly proved change in function potentials of temporal muscles. Additionally, their activity reaches the level of ca. 10% of masticators activity. In order to make a precise mapping of the biomechanical phenomena occurring at the time of incisors loading, cross-sectional area of the temporal muscle was taken at the level of 10% of the cross-sectional area masseter muscle. Such an assumption is justified from the physiological point of view, since growth in value of the recorded activity potential of the muscle is directly proportional to the force generated by the muscle. The schematic diagram, constituting a formal basis for bringing necessary analytical dependencies, which at the same time reflects the occurring relations between the skeletal system and the muscle system, is presented in Figure 1.

Bearing in mind the model assessment of the value of forces generated by adduction muscles of the mastication organ in various zones of the dental arch, the possibility of occlusion force displacement was considered *F*_*O*_. The parameter defining the place of the occlusion force in the formulated model is dimension *a*_*ZO*_. Points marked with symbols *E*_*i*_ (Figure 1) represent immobile muscles insertions to the cranial bones. The location of muscles insertion to the mandible is defined by polar coordinates, which are defined with the position vectors *OA*, *OB*, *OC*, and *OD* and angles *φ*_*M*_, *φ*_*P*_, *φ*_*L*_, and *φ*_*T*_. Lengths of particular muscles, included in computer simulations, are marked with symbols *L*_*i*_. The differential equation of the mandible movement constituting a formal basis for muscles forces identification takes the form of

(1) Jφ¨+FMRMLMhMcos⁡φM+φ+aMsinφM+φWW+FPRPLPhPcos⁡φP+φ+aPsinφP+φWW+FTRTLThTcos⁡φT−φ−aTsinφM−φWW−FLRLLLhLcos⁡φ:−φ+aLsinφL−φ=FOaZO.

**Figure 2:**
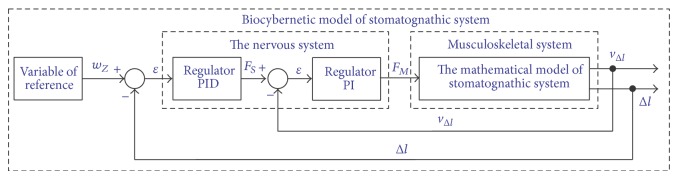
The generalised block diagram of muscle forces of the mastication organ identification.

Equation (1) is nonlinear owing to the geometry of placement of the muscle fibers. Mass moment of mandible inertia J may be estimated based on research results published in the work by Zhang et al. [14]. From the biocybernetic perspective the unknown muscle forces are calculated on the basis of the adopted control law. The basis for identification in the proposed method is comparison of the set size (control) *s*_*Z*_ with the regulated size *s*_*W*_. Additionally, comparison of these variables is possible as a result of applying negative feedback. General block diagram of muscle forces of the mastication organ identification in the biocybernetic perspective is presented in Figure 2.

The effect of the central nervous system (CNS) on muscles was reflected by applying regulators of continuous action, types PID and PI. The block diagram presented in Figure 2 has an additional loop of negative feedback applied. The initial computer simulation, based only on a single loop, would not provide satisfactory results. Lack of the assumed results was connected, first of all, with too great angle dislocation of the mandible, resulting from occlusion force. The application of an additional speed feedback loop substantially improved the quality of muscle forces identification. At this point it should be mentioned that the main task of the additional feedback is comparing the speed of shortening or extending muscle fibres. When formulating the final calculation model it should be determined which variables are adjustable sizes and which are control ones. In this paper regulated sizes *s*_*W*_ include changes in muscle length, while control sizes *s*_*Z*_ are forces generated by particular mastication organ muscles. Bearing in mind the adopted model assumptions, the control criterion was defined on the basis of diagram presented in Figure 2, whose general form is presented by the equation

(2) $$\begin{array}{c} \overset{\infty }{\underset{0}{\int }}\varepsilon \left(t\right)dt=0⟹\overset{\infty }{\underset{0}{\int }}\left(w_{Z}-\Delta l\right)dt=0. \end{array}$$

**Figure 3:**
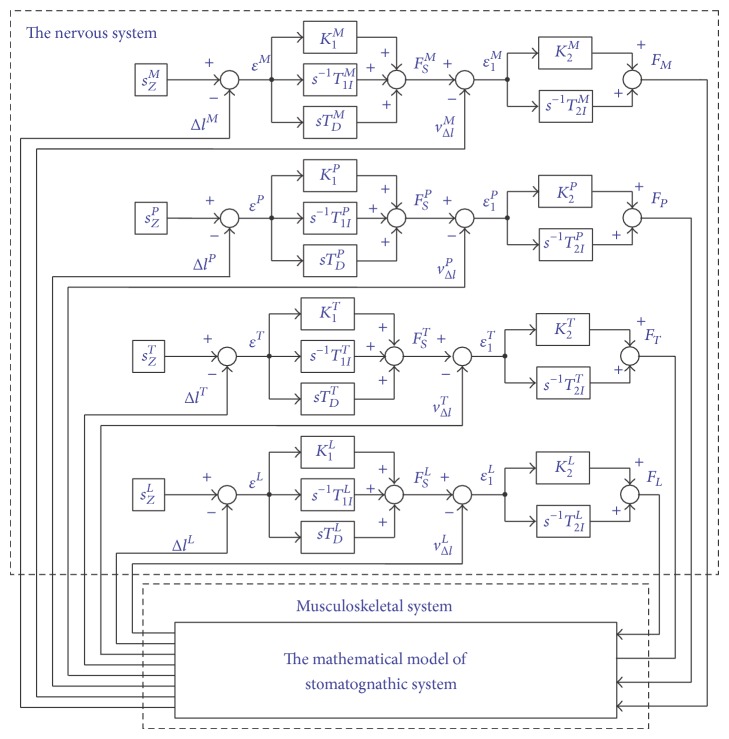
The generalised block diagram of muscle forces of the mastication organ identification.

The control criterion (2) demonstrates that, during loading the mandible with occlusion force, the length of the muscle cannot be changed, regardless of the value of the load exerted on the dental arch. If excessive occlusion load value results in mandible rotation, then there is no basis to state that the mandible remains in a biostatic balance. The analysed research task was formulated with the use of principles binding in issues concerning dynamics. In the formulated model, the mandible has the possibility of angular movement in relation to the axis running through the centres of condyloid process heads. Additionally, the movement must be compensated by regulators; otherwise, the analysed system becomes unstable. A detailed block diagram of adduction muscle forces of the mastication organ identification, in the biocybernetic perspective, is presented in Figure 3, and superscripts correspond to muscles: *M* is* masticator*, *P* is* medial pterygoid*, *T* is* temporalis*, and *L* is* lateral pterygoid*.

Control laws brought out on the basis of a detailed block diagram (Figure 3) data are then the following equations:

(3)  εLs=sZLs−ΔlLs, FSLs=K1LεLs+T1ILεLss+sTDLεLs, ε1Ls=FSLs−vΔlLs, FLs=K2L·ε1Ls+T2ILε1Lss, εPs=sZPs−ΔlPs, FSPs=K1PεPs+T1IPεPss+sTDPεPs, ε1Ps=FSPs−vΔlPs, FPs=K2Pε1Ps+T2IPε1Pss, εTs=sZTs−ΔlTs, FSTs=K1TεTs+T1ITεTss+sTDTεTs, ε1Ts=FSTs−vΔlTs, FTs=K2Tε1Ts+T2ITε1Tss, εMs=sZMs−ΔlMs, FSMs=K1MεMs+T1IMεMss+sTDMεMs, ε1Ms=FSMs−vΔlMs, FMs=K2Mε1Ms+T2IMε1Mss.

The included block diagrams and analytical dependencies from (1) to (3) are the formal basis for conducting necessary computer simulation, taking into account the effects of the central nervous system on forces generated by human masticatory system adduction muscles.

**Figure 4:**
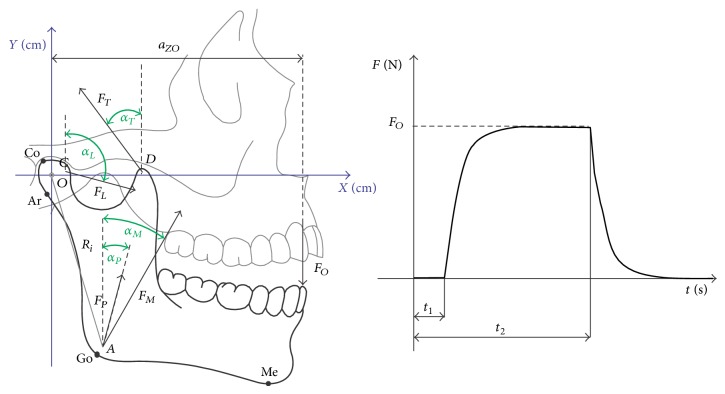
The diagram showing geometric structure of the mandible, along with marked places of mandible load with muscle forces and temporary characteristics of occlusion force accumulation.

Identification of muscle forces was conducted for three mandible models which correspond to the following faces: short, normal, and long. The anterior lower face height to the anterior total face height ratio was used for assignment of the subjects as either SF, normal, or LF. The rationale behind the use of this ratio is its strong predictive power of vertical craniofacial morphology as has been established by the use of discriminant analyses. Following other studies of LF and SF morphology, 79 and 80 subjects with a ratio exceeding 59.0% were considered to have a LF, whereas subjects with a ratio smaller than 54.0% were labeled as SF, although selection of “golden” cut-off points is inevitably arbitrary. Each model was analysed, for mandible load cases with force *F*_*O*_, in the area of incisors and molars. Additionally, the area of dental arch load has been obtained by changing the parameter value *a*_*ZO*_. The diagram showing distribution of active forces, operating on the mandible and points describing its geometric structure, is presented in Figure 4.

Moments of time are marked in Figure 4 with symbols meaning *t*_1_ the moment of loading the mandibular dental arch with force *F*_*O*_. However, time *t*_2_ defines the moment when the mandibular dental arch is relieved. Model assumptions as well as the adopted, idealized profile of occlusion force accumulation are an integral part of research in terms of muscle forces identification. Such an idealized nature of occlusion force increase was adopted for model tests, because it shows similarity to occlusion forces recorded with the use of an electronic dynamometer. However, in order to perform any model test, it is essential to have knowledge of specific numeric data. In this paper the required data in particular concerning the directions of muscle forces operation, muscles cross sections, and geometrical structure of the mandible were taken from the works by van Spronsen, 2010, and van Spronsen et al., 1996 [15, 16].  Table 1 compares parameters describing the adduction muscles and geometric construction of the studied craniofacial types. The numeric data are necessary to conduct a computer simulation concerning muscle forces identification in the biocybernetic perspective. In addition, an extremely significant biophysical size, defining the muscle, is the coefficient of unit efficiency of cross section, *k*. The research reports—Weijs and Hillen, 1985; Peck et al., 2000 [10, 17]—indicate that the value of this coefficient is within the scope from 30 to 40 N/cm^2^. Additionally, such a discrepancy in values has a decisive impact on the final effect of identification. This happens because the product of cross section A and coefficient *k* determines the maximum value of force generated by a given muscle. For the purpose of model tests in this paper, the maximum efficiency of particular adduction muscles has been estimated assuming *k* = 35 N/cm^2^.

Symbols presented in Table 1 are as follows: *R*_*i*_ are the place of muscle forces that is defined by position vectors *OA*, *OB*, *OC*, and *OD* (Figure 1). Parameters *φ*_*i*_ define the orientation of position vectors *R*_*i*_ in an arrow plane and are measured as presented in Figure 1; *α*_*i*_ defines the orientation of muscle forces identification in the arrow plane (Figure 4). While* Area* represents cross-sectional areas of muscles, *F*_MAX_ maximum forces, particular muscles, can generate. Change in forces generated by the mastication organ muscles is characterised by a nondimensional muscles activity coefficient *w*_*A*_. In this study, these coefficients are defined as the relation of force generated by a given muscle to the sum of forces generated by all the muscles. Analytical dependencies, characterizing activity coefficients of particular muscles, which have been included in computer simulations, are expressed by the equations

(4)  wAMasseter=FMFT+FM+FP+FL, wATemporalis=FTFT+FM+FP+FL, wAMedial  pterygoid=FPFT+FM+FP+FL, wALateral  pterygoid=FMFT+FM+FP+FL,

where *F*_*M*_ is masticator, *F*_*T*_ is temporalis, *F*_*P*_ is medial pterygoid, and *F*_*L*_ is lateral pterygoid.

## 3. Model Test Results

**Figure 5:**
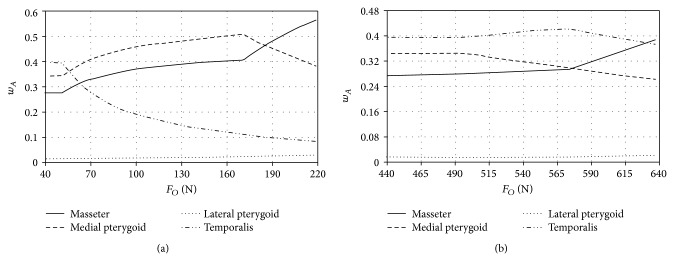
The activity of adduction muscles of the mastication organ of short craniofacial morphology during loading the mandibular dental arch in the area (a) of incisors and (b) of molars.

**Figure 6:**
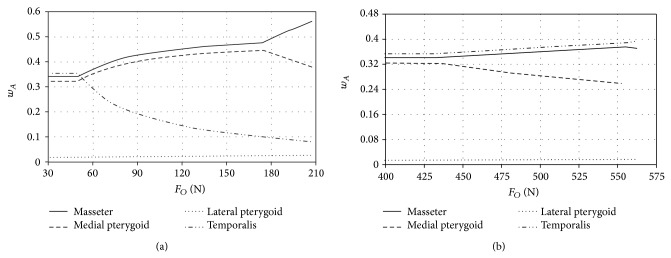
The activity of adduction muscles of the mastication organ of normal craniofacial morphology during loading the mandibular dental arch in the area (a) of incisors and (b) of molars.

**Figure 7:**
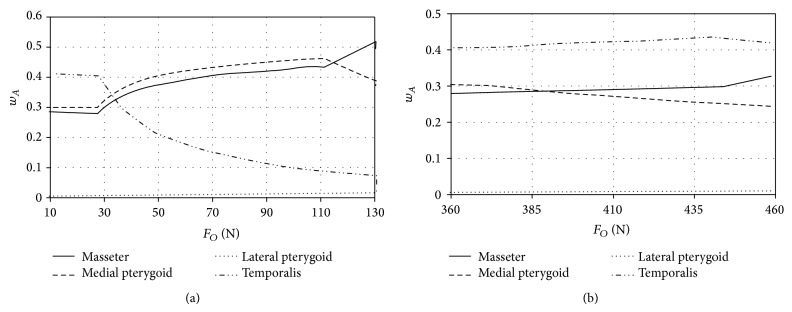
The activity of adduction muscles of the mastication organ of long craniofacial morphology during loading the mandibular dental arch in the area (a) of incisors and (b) of molars.

Model test results, which illustrate the change in muscles activity during loading the mandibular dental arch with occlusion forces, located in the zone of incisors and molars are presented in the charts (Figures 5, 6, and 7). At this point it should be expressly stated that the maximum values of forces shown in the charts are identical with the end of numeric calculations, namely, achieving the maximum efficiency by all the muscles included in computer simulations.

**Figure 8:**
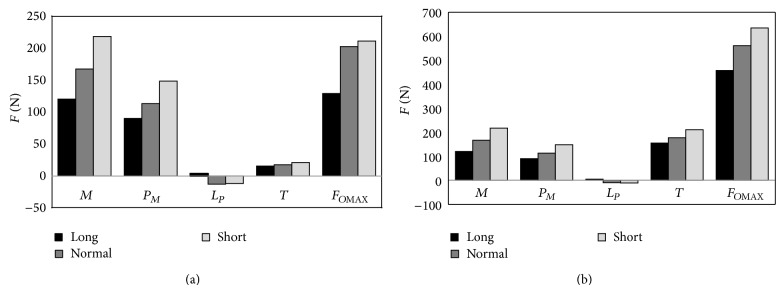
Force generated by the adduction muscles of the mastication organ during loading the mandibular dental arch with the maximum force in the area (a) of incisors and (b) of molars.

The next stage of model tests was estimation of maximum occlusion forces in the area of incisors and molars, which are caused by the activity of mastication organ muscles. Defined in such a manner research problem was solved in the way that calculation models, characterizing averaged mandibles of three types of anatomical craniofacial construction, were loaded with force *F*_*O*_ until achieving the border of system stability. The maximum value of forces *F*_*O*_, which did not cause loss of calculation model stability, was considered the maximum occlusion force, generated by the mastication organ muscles in the set dental arch area. The results of computer simulation are graphically shown in Figure 8.

Symbols shown in Figure 8 represent the following muscles: *M* is masticator, *P* is medial pterygoid, *L* is lateral pterygoid, and *T* is temporalis. The conducted computer simulations assumed that the boundary value of angular mandible movement cannot exceed the value of 0.2°. However, regulation errors, determining the change in muscles length, were lower than 0.01 mm. Anatomical data concerning average values of the cross-sectional areas of muscles, their orientation, and geometrical structure of the mandible (Table 1) were the formal basis for conducting necessary computer simulation. The numerically calculated occlusion forces, loading the mandibular dental arch in the area of incisors, in the case of short face reached the value of 636.61 N, normal 562.4 N, and long 458.56 N. The calculated maximum values of occlusion forces, generated in the zone of incisors, amounted to 218.4 N for short face, 207.1 N for normal face, and 130.4 N for long face.

## 4. Discussion

The operation of the central nervous system is the basic element ensuring correct functioning of mastication organ muscles. Optimum muscular action is not possible without bioelectric stimulation, whose source is the nervous system. For this reason, when making attempts of numeric modelling of living organism's movement as well as during muscle forces identification, each time an account should be taken of causal relations occurring between the muscular-skeletal system and the nervous system. Test results obtained this way provide qualitatively new information on the functioning of living organism's muscles. An analysis of complex processes taking place within the stomatognathic system using classical methods is determined, above all, by difficulties, related to muscle forces identification. One of the ways of mathematical projection of the impact of the central nervous system on the muscles is the use during model tests optimisation methods: Osborn and Baragar, 1985 [3], and Trainor et al. [18]. From the point of view of biomechanics, one of the most important parameters, typical for muscles, is information concerning the maximum force generated by these muscles. Final data are required for that, concerning the cross-sectional size of the studied muscle A. The confirmation of this statement is in many publications on this issue: Hsu et al., 2001; Goto et al., 2002; Gionhaku and Lowe, 1989; Kitai et al., 2002; and van Spronsen et al., 1989, 1992 [19–23]. Based on data contained in works mentioned above, it is possible to determine the average values of cross sections of the stomatognathic system muscles and then define their maximum efficiency.

Model tests of biostatic balance of the human mastication organ included in this work focused on two issues. The first one was to determine characteristics describing the variability of activity coefficients *w*_*A*_ of particular mastication organ muscles depending on the occlusion force *F*_*O*_. The second one focused on determining the maximum occlusion force possible to be generated by the stomatognathic system muscles in the area of incisors and molars. On the basis of the prepared characteristics describing the variability of activity coefficients of muscles *w*_*A*_ it can be stated that, regardless of craniofacial anatomical construction as well as the place of occlusion forces, the greatest activity in the initial phase of loading is shown by the temporal muscles (Figures 5 to 7). Subsequently there are medial pterygoid muscles and the lateral pterygoid muscles as the last ones. Only in the case of normal craniofacial anatomical construction do masticators generate greater forces as compared with medial pterygoids. In the case of the analysed normal face model this is connected with the average directions of *α*_*i*_ muscle forces actions (Table 1).

According to Ide and Nakazawa [13] occlusion load located in the area of incisors is caused mainly by simultaneous activity of masticators, medial pterygoids, and lateral pterygoids. Ide and Nakazawa state that, during generating loads in the area of incisors, fibres of the temporal muscles do not operate. Additionally, the function potentials measurements, conducted by the authors of the paper, that were recorded with surface electrodes placed above the front fibres of the temporal muscles show almost ten times lower activity as compared with surface masticators fibres. The growing occlusion load operating in the area of incisors results in the fact that, in the first place, the maximum efficiency is achieved by the temporal muscles (Figures 5(a) to 7(a)). From that moment growth is observed in masticators activity and medial pterygoids activity. This situation lasts until medial pterygoids reach the limit value of their efficiency. After this time only the activity of masticators and lateral pterygoids increases. Additionally, such a condition is maintained until masticators reach their maximum efficiency and then rapid growth in activity of lateral pterygoids is observed. When occlusion forces are generated in the area of molars (Figures 5(b) to 7(b)) in the initial phase of loading the mandibular dental arch, the greatest activity is observed in the case of the temporal muscles. However, maximum efficiency is achieved by the medial pterygoids. The mechanism of mastication organ muscles functioning, at occlusion forces, located in the area of molars is analogous to the case of occlusion forces interacting in the area of incisors. The obtained model tests' results indicate that the maximum occlusion forces generated in the area of incisors are mainly the result of masticators activity and medial pterygoids activity. The importance of temporal muscles becomes evident when the forces loading the dental arch move towards the molars (Figure 8). In the case of craniofacial morphology of short anatomical construction, occlusion forces generated by masticators in the area of molars are the largest (Figure 5(b)).

Achieving maximum efficiency by one of the muscles directly causes changing the value of activity coefficients of the other muscles. In other words, if a given muscle reaches maximum efficiency, then its activity is limited at simultaneous growth in activity of other muscles. At this point, it is worth mentioning that the obtained results of the computer simulation indicate that, regardless of the load area of the dental arch and craniofacial type, the lateral pterygoids demonstrate low activity. The activity of these muscles is strictly related to the role they perform in the functioning of the stomatognathic system. The fact that lateral pterygoids are mainly responsible for pulling out the mandible and they support the suprahyoid muscles during abduction [24] is also worth mentioning. In addition, it should be remembered that ca. 30–40% of the area of lateral pterygoids muscle fibres insertion is located on transport disks. Therefore, one of their tasks is disks movement in accordance with the condyloid process movement. Thus, it is reasonable to claim that lateral pterygoids do not play any key role in issues related to the biostatic balance of the mastication organ. The statement seems to be contradictory in relation to the size of lateral pterygoid cross section of the casement, which would indicate its substantial efficiency. However, from the point of view of biomechanics, and particularly directions defining the courses of muscle fibres and location of insertions to the mandible, its meaning and role are generally insignificant in issues concerning biostatic balance of the mastication organ. Clearly, observations made by Ide and Nakazawa [13] indicate that symmetrical load of the mandibular dental arch in the area of molars is a result of intensive activity of most mastication organ muscles, except the lateral pterygoids, which demonstrate relatively low activity.

Regardless of the load area of the mandibular dental arch, it has been stated that the largest values of occlusion forces are observed in people with short face, while the lowest with long craniofacial anatomical construction. Bearing in mind the fact that so far there has been no technology enabling direct measurement of forces generated by muscles, other methods of verification of model tests' results should be applied. One indirect way which enables result evaluation of computer simulation is clinical tests in which occlusion forces are recorded. In these tests specialised dental dynamometers are implemented and it is possible to single out two basic constructions of measuring devices. The first group includes dynamometers where occlusion forces are determined on the basis of plastic deformation size of the measuring sample. This method of occlusion forces measurement was described in detail in the work by Chladek et al., 2001 [25]. The second group are dynamometers where the force is recorded by various types of sensors, among others, pressure, Braun et al., 1995; Kamegai et al., 2005 [26, 27], or piezoresistive, Flanagan et al., 2012 [28]. Adduction muscles of the human mastication organ, in the area of molars, are able to generate loads, which can exceed the value of even 1 kN according to Braun et al., 1995, and Ferrario et al., 2004 [26, 29]. However, values of this range are found in few people, wherein the values of this order can generate a few individuals. Average values of occlusion forces recorded in the area of molars are in the scope from 300 up to 600 N in the case of women and from 400 to 700 N in the case of men, which was shown in works by Bakke et al., 1990; Braun et al., 1995 [26, 30]. Along with relocation of measurement point towards incisors, decrease in the measured occlusion forces is observed for the value of ca. 100 N in the case of women and ca. 200 N in the case of men, which is confirmed by the following researches: Paphangkorakit and Osborn, 1997, 1998; Regalo et al., 2008 [31–33]. It should also be borne in mind that, along with general ageing of the body, occlusion forces generated by adduction muscles of the mastication organ are limited, Bakke et al., 1990 [30]. It is believed that this phenomenon is caused mainly by loss of muscle fibres, Faulkner et al., 2007 [34].

In order to verify the model tests' results obtained in this paper, the results of computer simulation referred to results published in the work by Abu Alhaija et al., 2010 [35]. Average values of occlusion forces were recorded in the area of molars in a group of 30 women and 30 men aged ca. 22. Among the focus group three subgroups with different craniofacial construction were selected. Occlusion forces were recorded by means of an electronic dental dynamometer GM10, obtaining average values in the area of molars for short face 679.6 N, normal face 593.08 N, and long face 453.57 N. Numerically calculated by the authors of this study, occlusion forces amounted to, accordingly, in the case of short faces 636.61 N, normal 562.4 N, and long 458.56 N. High compliance of the results obtained in the research model relative to the average forces recorded in the clinical trials was found. Clearly, relative errors of occlusion forces, obtained in the model tests and clinical tests, were for short face 6.33%, normal face 5.17%, and long face 1.10%.

In the event when the stomatognathic system muscles generated load in the area of incisors, the maximum occlusion forces amounted to 218.4 N for short face, 207.1 N for normal face, and 130.4 N for long face. It is impossible for the results of these model tests to relate to specific clinical tests that were conducted on various craniofacial types as in the case of occlusion forces generated in the area of molars. However, the works by Paphangkorakit and Osborn, 1997, 1998; Regalo et al., 2008 [31–33], show that occlusion forces recorded in the area of incisors are within the scope from 120 N to 225 N in men and from 64 N to 185 N in women. The values are similar to the results obtained on the basis of the conduced computer simulation.

This kind of numeric considerations seems to be also important from the point of view of function disorders in diagnostics of the mastication organ movement system especially the temporomandibular joint dysfunctions [36–39]. It is known that patients suffering from bruxism [40] are able to generate occlusion forces often exceeding the value of 1 kN. Face type, short, normal, or long, may be a genetic feature but may equally well result from orthognathic or occlusion defects and such factors can significantly affect the length of the lower part of face, determining its comprehensive dimension. Vertical dimension of the face is important in issues of compressive forces exerted on articular structure, which was demonstrated by van Spronsen et al. [41]. In other words, elimination of occlusion defects, especially in vertical dimension, may contribute to change in face qualification from short, for example, to normal, which, in turn, affects the reduction mastication muscles forces, which can have destructive impact on particular elements of the stomatognathic system, mainly joints. The conducted research partially also seems to explain the importance of loss of occlusion height. Quality shortages of the dental hard tissues, pathological abrasion, untreated dental caries, or iatrogenic actions can affect normal face transformation to be short, which leads to increase in forces generated by muscles with all the negative effects.

## 5. Conclusions

To sum up, the method presented in this paper should be considered an alternative approach to identifying muscle forces and model evaluation of occlusion forces generated by adduction muscles of the mastication organ. The proposed method treats muscles as “black boxes” whose properties independently adapt to external conditions. On the basis of the conducted computer simulation it is possible to formulate the following general applications.

1. Considering the limitations of the maximum force generated by particular muscles provides qualitatively and quantitatively new information about functioning of the masticatory muscels.
2. Model assessment of occlusion forces generated by muscles and identification of the mastication organ adduction muscles forces may constitute an important tool in diagnostics of the mastication organ dysfunctions, especially related to vertical occlusion defects.

During identification of the mastication organ muscles forces it is not required to know the mathematical model of the muscle as well as to define the degree of its activity. The conducted model tests assume the criterion of constant value regulation. However, it should be expressly pointed out that it is possible to adopt control law reflecting the variable value regulation. Assuming such control laws, it is possible to identify forces generated by the mastication organ muscles in operation: mandible protrusion or abduction and in the action of chewing.

## Figures and Tables

**Table 1:** Numeric data adopted for the need to conduct numeric calculations.

	Muscle name	*R*_*i*_	*φ*_*i*_	Area	*F*_MAX_	*α*_*i*_	Ar-Go	Go-Me	Co-Me	<Go
		[cm]	[°]	[cm^2^]	[N]	[°]	[cm]	[cm]	[cm]	[°]
Short face	Masticator	5.97	−77	6.22	217.7	−8	5.64	7.64	12.06	116
	Medial pterygoid	5.97	−77	4.23	148	−13				
	Lateral pterygoid	0.30	15	4.31	150.8	−97				
	Temporalis	3.53	4	6.02	210.7	27				

Normal face	Masticator	5.71	−75	4.77	166.9	−19	5.70	7.71	12.39	125
	Medial pterygoid	5.71	−75	3.23	113	−17				
	Lateral pterygoid	0.32	22	4.16	145.6	−97				
	Temporalis	3.64	2	5.07	177.4	27				

Long face	Masticator	5.37	−83	3.43	120	−17	4.61	7.43	12.15	134
	Medial pterygoid	5.37	−83	2.57	89.9	−18				
	Lateral pterygoid	0.52	−16	3.77	131.9	−99				
	Temporalis	3.85	−8	4.43	155	27				
